# Food systems for resilient futures

**DOI:** 10.1007/s12571-020-01070-7

**Published:** 2020-07-10

**Authors:** Helena Kahiluoto

**Affiliations:** Sustainability Science, LUT University, Mukkulankatu 19, 15210 Lahti, Finland

**Keywords:** Assessment, Determinant, Efficiency, Resilience, Response diversity, Scenarios

## Abstract

In this time of the pandemic, nothing is as it used to be. This change creates space for new narratives towards resilience. The resilience perspective implies preparing for shocks as well as various futures that might evolve. Thus, more sustainable food systems cannot only be built to be pandemic proof. This preparation can be facilitated by co-designing contrasting future narratives, identifying means for developing capacity to adapt to those futures and developing tools to enhance that capacity, such as demonstrated here. The capacity of food systems to adapt and transform is enhanced by dialogue, transparency and collective learning in food value chains and networks, sovereignty over resources, and built-in diversity in response to change. In market-led global food chains, supplier-buyer diversity is important, while in public-led regions with some market protection, farm and crop diversity might matter more in response to variability in weather, price and policies. During, for example, an international conflict, or the time of a pandemic, diverse food sourcing from local producer-consumer cooperatives to community-supported and urban agriculture could secure food for citizens. Assessments of critical diversity in response to shocks and volatility can help actors to tailor effective diversity to manage resilience while avoiding the long-feared trade-off between diversity and resource-use efficiency. The interdependence of humanity deserves attention, as food systems are only as resilient as their weakest actor. A truly resilient global food system implies not only preparedness for coming shocks and changes but also a foundation that makes shocks less probable and critical.

## COVID-19 opens food systems up for a change

In the time of the COVID-19 pandemic, nothing is as it used to be, and so it may continue. This change creates space for new narratives of the future and the demand for new tools to achieve such narratives. Triggered by a crisis, the operational environment or ‘regime’, opens up, creating windows of opportunity for novelty (Kuokkanen et al. 2018), and diversity and variation of initiatives provide material for selection (Geels 2002; Geels and Schot 2007). For food systems, the crisis enabling such a regime shift could be, for example, a food crisis with price peaks (Tadesse et al. 2016; Kahiluoto et al. 2020), terrorism, conflict, climate change or an epidemic. This pandemic has created both a demand and the momentum to transform to more resilient food systems.

Resilience implies the capacity to maintain the core function of a system when facing volatility or unpredictable change. Thus, a resilient food system would provide food and nutrition security to all in all plausible conditions. Resilience at all levels is required because the future is uncertain, and the next, still unknown, shock may be just around the corner. Therefore, new, more sustainable food systems cannot only be built to be pandemic proof. Much of the resilience literature has focused on developing theory or resilience ‘thinking’. Indeed, adopting this systems-oriented viewpoint of sustainability, in complement to resource-use efficiency (Goerner et al. 2009; Korhonen and Seager 2008; Ulanowicz et al. 2009), is an important starting point for making food systems more robust to disturbances and a step-by-step exercise to resilience thinking has been shown to make a difference in actors’ perspectives (Himanen et al. 2016). Fortunately, attempts to empirically identify and test the determinants of resilience have also emerged. Such determinants can help actors in food value chains, as well as administrators and policymakers, to enhance resilience at various levels of food systems. In fact, if resilience is not explicitly considered, a decline in resilience might take place, as shown for European wheat (Kahiluoto et al. 2019a, b), implying severe threats to food security.

## Resilience is preparedness for many futures

The resilience perspective implies preparing for different plausible situations in the future. Different contexts may appear different times – like the pandemic now all over the globe – or in different parts of the world – such as droughts and yield failures year after year in Syria before the war. Yield failures, expansion of dedicated energy crops on agricultural land and speculation triggered the food crisis (Tadesse et al. 2016) and finally the emigration of more than five million people towards Europe in 2016. Even if the COVID-19 pandemic once again revealed the global interdependence, it also triggered local connectivity and attempts to national sovereignty led by public actors. The strategy for achieving resilience is different between the contrasting futures. In the market-led food value chain, trust may be based on contracts and brands, whereas public leadership emphasizes rules and authority, and civil society relies on social bonds and proximity (Kumar 1996). Diversity not only provides material for learning and transformation but also buffers against disturbance. In market-led global food systems, it is important to develop supplier-buyer diversity (Kahiluoto et al. 2020), while in public-led, partially protected regions such as the EU, encouraging farm and crop diversity in response to weather, price and policies might matter more. In local civil society, diverse market channels from cooperatives to community-supported and urban agriculture may provide security to citizens.

## The determinants of resilience provide means to management

Preparation to uncertainty can be aided by co-designing contrasting narratives of possible futures and the means to adapt to each. We engaged food system actors in an iterative, three-step co-creation exercise to identify means for building more resilient food systems. First, key actors of the Finnish food value chain were in a workshop in 2011 familiarized with imaginary shocks ranging from soaring energy prices to the vast immigration of people with very different diets, as well as with contrasting future food system scenarios varying the leading societal actor and values (Himanen et al. 2016; Kumar 1996). Thereafter, in a two-stage Delphi study, the actors considered what kinds of features and means would make the food system perform well in all these different, rapidly emerging conditions. The following major determinants of resilience were identified: (1) fair dialogue and collective learning across the food value chain, (2) sovereignty regarding key resources and (3) different kinds of diversity.

Dialogue, market transparency and symmetric information for all actors allow trust and commitment and thus the capacity to rapidly adapt the entire food chain to changes (Himanen et al. 2016). Collective learning requires innovativeness and experimentation. The importance of resource sovereignty to protect against price volatility and supply interruptions was particularly identified by the Finnish food system actors to concern energy sources and nutrient inputs. The availability and price of land is also increasingly faced as a bottleneck, not only in Finland but also, for example, in Russia and Africa, and the critical role of access to labour has been revealed by the COVID-19 pandemic.

## Assessments of response diversity as a tool to manage resilience

**Fig. 1:**
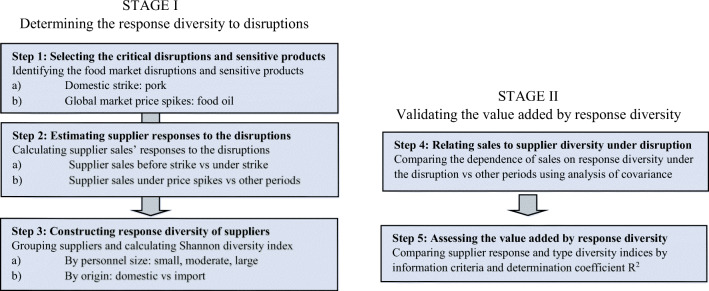
The proposed response diversity assessment for the management of the supply chain resilience (Kahiluoto et al. 2020). The generic approach can be applied at any other part of a food system as well from a field (cultivar or crop diversity, Kahiluoto et al. 2014a, Kahiluoto et al. 2019a, b; Mäkinen et al. 2015) to farm (land use or income source diversity, Kahiluoto and Kaseva 2016) and beyond

Diversity represents material for robustness, but also for collective learning and transformation. Polycentric governance provides experimentation to learn for new challenges, not least for climate change (Ostrom 2010). Sovereignty depends on diversity, for example, in terms of different options for replacing an abruptly unavailable resource, buyer or supplier (Kahiluoto et al. 2020). Diversity is indeed an important determinant of resilience in various parts of food system: diversity in protein sources including, e.g., legumes, insects, algae and microbes to prepare for land scarcity or climate change, diversity in income sources or land-use types on farms to protect against price volatility (Abson et al. 2013; Kahiluoto and Kaseva 2016), diversity of crop species and cultivars to address weather anomalies (Kahiluoto et al. 2014a, 2019a, b; Mäkinen et al. 2015), or genetic diversity of production animals in response to epidemics. Diversity per se (Page 2010) does not, however, necessarily enhance resilience, whereas diversity in response to shocks and change, i.e., response diversity, can maintain the system function in turbulent times (Elmqvist et al. 2003; Leslie and McCabe 2013; Kahiluoto et al. 2014a). If the key components of diversity that foster resilience are identified, more resilience can be achieved. A five-step generic approach to identify and manage the effective diversity can guide in building resilience from the field to the food chain, and beyond (Fig. 1).

The supplying of food is the core function of food systems, and stability during disruptions and volatility as well as adaptability are of primary importance to supply chain actors and societies (Stone and Rahimifard 2018). Supply disruptions can be local or domestic such as due to weather extremes or strikes, or they can be global, caused by global market price anomalies, conflicts in trade policies, abrupt immigration affecting food demand, or by pandemics. What kind of suppliers and buyers respond different ways to the most critical disruptions and thus stabilize supply and demand, can be empirically assessed. The generic approach to managing resilience through response diversity in food chains was demonstrated by considering the maintenance of retail store sales when facing a strike in the food industry and price anomalies in the global food market (Kahiluoto et al. 2020) (Fig. 1).

In the above-mentioned study, the following research question was posed: Is the response diversity of suppliers more positively associated with supply chain resilience than mere supplier diversity? Resilience was operationalized as maintaining sales of two food products in 27 southern Finnish retail stores during the two types of disruptions. Response diversity was operationalized as 1) diversity in the number of employees at the slaughterhouse suppliers of pork under a domestic strike and as 2) balance in the proportion of imports and domestic supply of food oil under global price volatility. As the result of the five-step quantitative assessment, response diversity was found to be more positively related to the maintenance of sales than the diversity of individual suppliers. Empirical assessments of the response diversity of suppliers provide buyer companies with an effective means to enhance their supply base management for resilience, and administrators with an option to enhance social security. The applicability of the assessment has rapidly increased along with the expansion of access to big data.

## Resilience and efficiency – Is there a trade-off?

While actors of the Finnish food system identified diversity as an important aspect of facilitating a successful response to various changes, many also mentioned the concern that diversity could make the supply chain and farms inefficient (Himanen et al. 2016). Until recently, this concern has been shared by a vast majority of decision-makers in agrifood systems across the globe. In recent decades, food supply chain efficiency has been streamlined for expected conditions, which in many cases has led to less diversity. In particular, such a paradigm has been dominant in industrial countries, where failures and uncertainty have not been part of daily life on the farm and at the dinner table for more than half of the century. Streamlining the supply chain enhances efficiency in stable conditions but might reduce the efficiency or even disable the function of a supply chain when facing the unexpected, such as demonstrated by supply disruptions in some lines of business relying solely on Chinese suppliers at initial stages of the COVID-19 pandemic.

Diversity is known to increase the stability of production in agricultural environments (Tilman et al. 2006), and land-use diversity appears to increase farm resilience in terms of economic returns (Abson et al. 2013). Empirical evidence for the dependence between resource-use (or economic) efficiency and the diversity of production, however, ranges from scarce to non-existent. The assumed trade-off between diversity and efficiency in real life appears to most often be avoided by managing a system such as a farm so that, for example, diversity in income sources does not cause labour peaks but rather temporally distributes the workload. This was concluded by a quantitative assessment of the dependence of resource-use efficiency and land-use diversity of Finnish farms where no indication of a trade-off was found (Kahiluoto and Kaseva 2016). Applying diversity in response to plausible changes, rather than diversity for its own sake, further increases the efficiency of diversity. The assumed knowledge that a trade-off exists between economic efficiency and diversity still informs agricultural policies and supply chain actors, and more research on this topic may be needed.

## Bringing food system resilience to the next level

Finally, the interdependence of humanity on a globe characterized by increasing scarcity deserves attention; food systems are only as resilient as their weakest actor (Adger et al. 2009; O'Brien et al. 2009). A truly resilient global food system implies not only preparedness for coming shocks and changes but also a foundation that makes shocks less probable and less critical. This requires food and nutrition to be provided to all, and within the carrying capacity of the planet. Loss and fragmentation of natural ecosystems was a driver of COVID-19 pandemic. Also dietary changes, waste reduction, circular technologies and redistribution of global resources need to be adopted (Kahiluoto et al. 2014b; Kahiluoto et al. 2015; Springmann et al. 2018), incentivized by new rules of the game. Fairness is not solely distributional, but just procedures and a culture with dignity are important pillars. The fear of being seen of less value creates violence (Wilkinson and Pickett 2011), and hunger represents extreme humiliation.

Could a new social contract (O'Brien et al. 2009) emerge after this most recent shocking reminder of the deep interrelatedness across humanity – similar to that emerging after World War II – and between the humanity and the nature? The COVID-19 pandemic has brought us to a crossroads in a story with an open end. Today, humanity once again has the opportunity to choose routes to various futures. Could an awareness of global interdependence now emerge to enable us to build a more sustainable, fair and resilient world?
